# High-volume prostate biopsy core involvement is not associated with an increased risk of cancer recurrence following 5-fraction stereotactic body radiation therapy monotherapy

**DOI:** 10.1186/s13014-023-02397-z

**Published:** 2024-03-04

**Authors:** Jonathan W. Lischalk, Astrid Sanchez, Vianca F. Santos, Christopher Mendez, Meredith Akerman, Todd Carpenter, Moses Tam, David Byun, David R. Wise, Anand Mahadevan, Andrew Evans, William Huang, Aaron Katz, Herbert Lepor, Jonathan A. Haas

**Affiliations:** 1 Department of Radiation Oncology, Perlmutter Cancer Center at New York University Langone Hospital—Long Island, 150 Amsterdam Ave., New York, NY 10023 USA; 2 Department of Radiation Oncology, Perlmutter Cancer Center at New York University Langone Hospital—Long Island, Mineola, NY 11501 USA; 3 Division of Health Services Research, NYU Grossman Long Island School of Medicine, Mineola, NY 11501 USA; 4 Department of Radiation Oncology, Perlmutter Cancer Center at New York University Grossman School of Medicine, New York, NY 10017 USA; 5 Department of Medicine, Perlmutter Cancer Center at NYU Langone Medical Center, New York, NY 10017 USA; 6 Department of Radiation Oncology, Perlmutter Cancer Center at New York University Langone Hospital, New York, NY 10017 USA; 7 Department of Urology, Perlmutter Cancer Center at New York University Grossman School of Medicine, New York, NY 10017 USA; 8 Department of Urology, Perlmutter Cancer Center at New York University Langone Hospital—Long Island, Mineola, NY 11501 USA

**Keywords:** Prostate cancer, Biopsy core involvement, SBRT, Gleason score, PSA, High-volume core involvement

## Abstract

**Purpose:**

Percentage of positive cores involved on a systemic prostate biopsy has been established as a risk factor for adverse oncologic outcomes and is a National Comprehensive Cancer Network (NCCN) independent parameter for unfavorable intermediate-risk disease. Most data from a radiation standpoint was published in an era of conventional fractionation. We explore whether the higher biological dose delivered with SBRT can mitigate this risk factor.

**Methods:**

A large single institutional database was interrogated to identify all patients diagnosed with localized prostate cancer (PCa) treated with 5-fraction SBRT without ADT. Pathology results were reviewed to determine detailed core involvement as well as Gleason score (GS). High-volume biopsy core involvement was defined as ≥ 50%. Weighted Gleason core involvement was reviewed, giving higher weight to higher-grade cancer. The PSA kinetics and oncologic outcomes were analyzed for association with core involvement.

**Results:**

From 2009 to 2018, 1590 patients were identified who underwent SBRT for localized PCa. High-volume core involvement was a relatively rare event observed in 19% of our cohort, which was observed more in patients with small prostates (*p* < 0.0001) and/or intermediate-risk disease (*p* = 0.005). Higher PSA nadir was observed in those patients with low-volume core involvement within the intermediate-risk cohort (*p* = 0.004), which was confirmed when core involvement was analyzed as a continuous variable weighted by Gleason score (*p* = 0.049). High-volume core involvement was not associated with biochemical progression (*p* = 0.234).

**Conclusions:**

With a median follow-up of over 4 years, biochemical progression was not associated with pretreatment high-volume core involvement for patients treated with 5-fraction SBRT alone. In the era of prostate SBRT and MRI-directed prostate biopsies, the use of high-volume core involvement as an independent predictor of unfavorable intermediate risk disease should be revisited.

**Supplementary Information:**

The online version contains supplementary material available at 10.1186/s13014-023-02397-z.

## Introduction

Percentage of positive prostate biopsy cores is a known independent risk factor for PCa aggressiveness. This was demonstrated nearly two decades ago when D’Amico et al. revealed PCa biopsy core involvement of ≥ 50% was associated with a 10.4 × relative risk of prostate-specific mortality versus those with < 50% biopsy core involvement [1]. This pathologic factor was ultimately used to help differentiate more belligerent forms of intermediate-risk PCa [2]. Consequently, it is currently included within the NCCN guidelines as an independent factor for the diagnosis of unfavorable intermediate-risk PCa.

The majority of data exploring the association between prostate biopsy core involvement and radiotherapy outcomes was published in the era of conventional fractionation. In the modern era, ablative radiotherapy techniques (i.e. SBRT) have demonstrated comparative effectiveness relative to conventional fractionation [3, 4]. Much of the early interest in ultra-hypofractionated schedules in the treatment of PCa grew out of our understanding that prostatic adenocarcinoma has a far lower alpha/beta (~ 1.85) than previously hypothesized [5]. As such, SBRT of 40 Gy in five fractions can deliver a much higher biologically effective dose (BED) compared to dose escalated intensity modulated radiation therapy (IMRT) of 78 Gy in 39 fractions (213 vs. 162, respectively).

It may be postulated, a larger BED could lead to volumetric eradication of adenocarcinoma to a greater extent. A recent publication from Memorial Sloan Kettering demonstrated two year post-SBRT prostate biopsies yielded lower rates of positivity when SBRT doses ≥ 40 Gy in five fractions were utilized [6]. Moreover, those patients found to have a positive two-year post-SBRT biopsy were significantly more likely to develop a biochemical relapse at five years. As such, there appears to be a correlation between dose fractionation and pathological ablation resulting in long-term biochemical control.

In the era of SBRT, can a higher BED mitigate the historical negative prognostic implications of high-volume disease? Herein, we explore the association between the extent of pre-SBRT prostate biopsy core involvement with post-treatment PSA kinetics and oncologic outcomes.

## Materials and methods

### Patient eligibility and treatment

The local Institutional Review Board (Study # 00001269) approved this single institutional review of patients treated for PCa. Exclusion criteria for the specific investigation of PSA nadir outcomes was as follows: (1) received ADT as a component of treatment, (2) did not have a pathology report available, (3) never achieved a PSA nadir (i.e. PSA < 3 ng/mL), (4) demonstrated disease progression, and (5) less than 2 years of FU. Analysis of core involvement association with oncologic outcomes excluded (3) and (4) above. All patients underwent rigorous surgical pathologic review of the prostate biopsy specimen. Each prostate pathology report was independently reviewed by our team to document the number of cores that were sampled, location, and the Gleason score that was identified. This evaluation included adenocarcinoma core involvement, overall percentage core involvement, and specific GS for a given positive core (PC). All patients were evaluated by a radiation oncologist and deemed appropriate for definitive 5-fraction SBRT. All patients underwent computed tomography (CT)-based radiation treatment planning simulation. A prostate MRI was obtained in the majority of cases at the time of simulation. Patients underwent robotic SBRT with a clinical target volume (CTV) which included the entire prostate and proximal seminal vesicles. A 5 mm isometric expansion of the CTV with a tighter 3 mm posterior margin was used to create the PTV.

### Follow-up and statistical analysis

Patients were typically followed using serial PSA and clinical examination at 3-month intervals for the first year and subsequently every 6 to 12 months thereafter. PSA nadir was defined as the lowest post-SBRT PSA obtained after at least 2 years of FU. High-volume core involvement was defined in accordance with the NCCN definition of ≥ 50% biopsy core involvement with adenocarcinoma. Descriptive statistics (mean ± standard deviation or median [25th, 75th] percentiles for continuous variables; frequency and percent for categorical variables) were calculated for the overall sample for patient, tumor, and treatment characteristics. A graphical display of PSA nadir was constructed using boxplots for the overall sample as well as stratified by low-, intermediate-, and high-risk groups.

The association between PSA nadir and percentage of PC, continuous percentage of core involvement and age, and PSA and prostate CTV was assessed using Spearman correlation coefficients. Core involvement was then analyzed by giving higher weight to PC involvement of higher-grade grouping. For example, a single core of GS 10 was weighted 5 × a single core involved of GS 6. Grade group weighting was as follows: GS6 – 1x, GS7 – 2x, GS8 – 3x, GS9 – 4x, GS10 – 5×. Analysis of variance was used to assess the association between categorical variables such as PSA, GS, NCCN Risk and continuous percent core involvement. Percent core involvement was dichotomized as < 50% and ≥ 50%. The two groups were compared using the chi-square test or Fisher’s exact test for categorical variables, and the two-sample t-test or Mann–Whitney test for continuous variables.

Time-to-failure analysis (biochemical and/or radiological failures) was accomplished using standard methods of survival analysis, where the data were stratified by dichotomized percent PC (< 50% vs. ≥ 50%). Biochemical failure was defined using the Phoenix definition of > 2 ng/mL above nadir. In cases where the endpoint event, “failure”, had not yet occurred, the number of years until last FU was used and ‘censored’. The groups were compared using the log-rank test. Cox Proportional Hazards regression was used to determine whether weighted total PCs were associated with “time-to-failure” alone, and after adjusting for the possible confounding effect of prostate CTV. All analyses were performed for the overall sample, and separately by NCCN risk group. A result was considered statistically significant at *p* < 0.05. Analyses were performed using SAS version 9.4 (SAS institute Inc.,). A multivariable analysis was conducted controlling for age, initial PSA, GS, staging and prostate CTV.

## Results

### Patient, tumor, and treatment characteristics

From 2009 to 2018, 1,590 patients were identified with a median age of 66 years, who underwent robotic SBRT for localized PCa. The distribution of risk grouping was as follows: low (n = 474, 30%), intermediate (n = 1061, 67%), and high (n = 55, 3%). Of note, due to the exclusion of patients who received ADT, there were very few high-risk patients included in this analysis. Median pretreatment PSA for low-, intermediate-, and high-risk PCa was 5.4 ng/mL (IQR: 3.6 to 6.7 ng/mL), 6.6 ng/mL (IQR: 4.7 to 8.7 ng/mL), and 7.7 ng/mL (IQR: 5.5 to 10.1 ng/mL), respectively. The clinical stage distribution was predominantly T1 (n = 1269, 84.49%). All patients were treated with definitive robotic SBRT over five treatment fractions to a total dose of 3500 (n = 1451, 91.26%) or 3625 (n = 131, 8.23%) cGy. Of note, treatment dose information is unknown for eight patients (< 1%) due to the transition from physical to electronic records. Median prostate CTV was 77.61 cc (IQR: 61.3 to 99.2 cc) and was prescribed to a median isodose line of 84% (IQR: 83% to 85%). Detailed patient, tumor, and treatment characteristics are listed in Table 1.

**Table 1:** Patient, tumor, and treatment characteristics (n = 1590)

Median age (years)	66 [61, 71]
*Age (range, years)*	
< 60	331 (21%)
[60–70]	814 (51%)
> 70	445 (28%)
Median PSA (ng/mL)	6.0 [4.6, 8.0]
*PSA (ng/mL)*	
< 10	1353 (85%)
[10–20]	231 (14%)
> 20	6 (1%)
*Gleason Scores*	
6	546 (34%)
7	993 (62%)
8	45 (3%)
9	6 (1%)
*NCCN Risk*	
Low	474 (30%)
Intermediate	1061 (67%)
High	55 (3%)
*Total number cores*	
< 12	56 (4%)
12	1503 (94%)
> 12	31 (2%)
Median % core involvement	25.0 [16.7, 41.7]
*Core involvement*	
0–25	893 (56%)
26–50	535 (33%)
51–75	124 (7%)
75–100	38 (3%)
Median Prostate CTV (cc)	77.8 [61.3, 99.1]
*Prostate CTV (cc)*	
< 50	149 (10%)
50–100	878 (55%)
101–150	246 (16%)
151–200	36 (2%)
> 200	44 (3%)
Unknown	233 (15%)

Data reported as median [25th, 75th percentiles] for continuous measures and frequency (%) for categorical variables

**Fig. 1:**
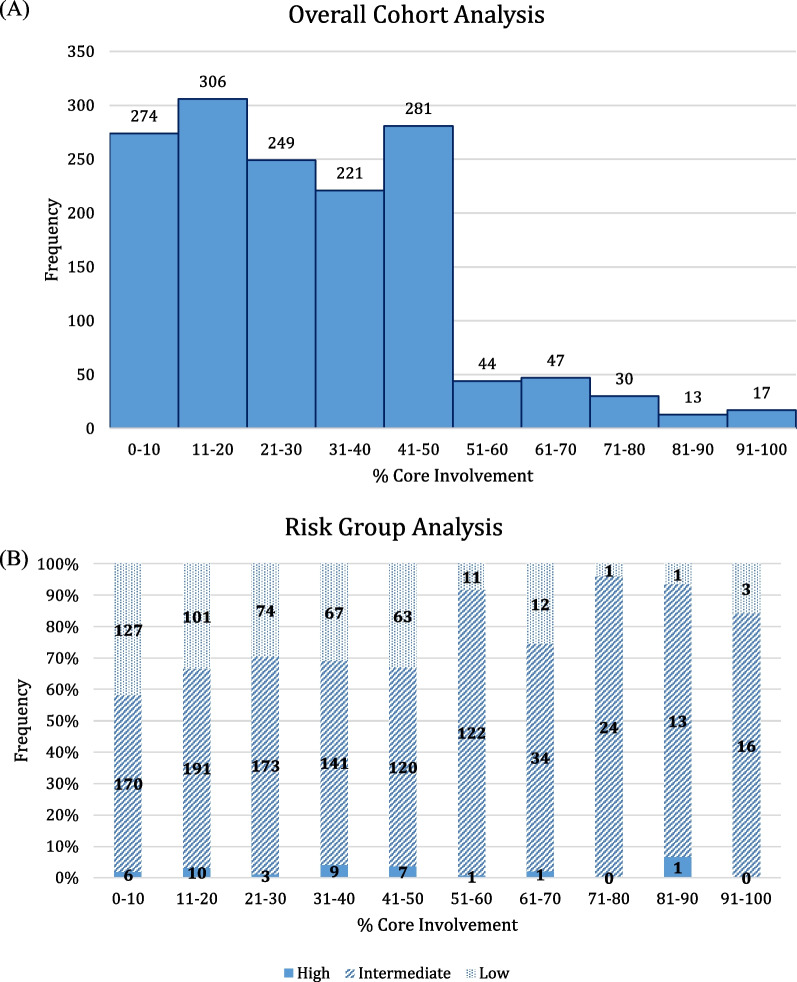
Histograms displaying core involvement for the (**A**) overall cohort, and (**B**) by risk factor

Surgical pathology review demonstrated an overall GS distribution as follows: GS6 (n = 546, 34%), GS7 (n = 993, 62%), GS8 (n = 45, 3%), and GS9 (n = 6, 1%). The median percent biopsy core involvement was 25% (IQR: 16.7% to 41.7%). Median percent core involvement for low-, intermediate-, and high-risk was as follows: 20.7% (IQR: 8.3 to 33.3), 25% (IQR: 16.7 to 41.7), and 33.3% (IQR: 16.7 to 41.7), respectively. Overall, high-volume pretreatment biopsy core involvement was uncommon in this cohort with only 19% of the entire group (n = 303) demonstrating this elevated burden of disease. Biopsy core involvement distribution is illustrated for the overall cohort and stratified by risk group in Fig. 1A-B.

### Clinical predictors of biopsy core involvement

Analysis of pretreatment clinical characteristics was performed to determine if there were any associations with high-volume biopsy core involvement (≥ 50%). When stratified by risk grouping, high-volume core involvement was observed in 14% of low- (66 of 474), 21% of intermediate- (226 of 1061), and 20% of high-risk (11 of 55) patients. Significantly more patients diagnosed with GS7 were found to have high-volume core involvement (73% vs. 60%, *p* = 0.0001). Similarly, significantly more patients with intermediate-risk disease were found to have high-volume core involvement compared to the rest of the cohort (75% vs. 65%, *p* = 0.005). In contrast, significantly fewer patients diagnosed with GS6 (25% vs. 38%, *p* = 0.0001) and high-risk disease (23% vs. 33%, *p* = 0.0053) were found to have high-volume core involvement. Finally, smaller prostate volume (PV), as defined by the CTV, was associated with high-volume core involvement (70 cc vs. 79 cc, *p* < 0.0001). There was no significant difference in high-volume core involvement based on patient age or initial PSA, analyzed either as a continuous or discrete variable. Detailed analysis on the association between core involvement and patient characteristics are found within the Additional file 1, 2, 3, 4, 5: Tables 1A, 1B, 1C, 1D, 1E.

Biopsy core involvement was also analyzed as a continuous variable rather than using a discrete 50% breakpoint. Analogously, we observed intermediate-risk disease to display a significantly higher percent core involvement (32.0% ± 19.7%) relative to low- (30.0% ± 17.3%) and high-risk (25.6% ± 17.0%) disease (*p* < 0.0001). Furthermore, prostate CTV displayed a significant negative correlation with biopsy core involvement (ρ = − 0.14, *p* < 0.0001). Again, there was no association with patient age (*p* = 0.95) or pretreatment PSA (*p* = 0.57) (Supplementary Table 1E).

### Core involvement association with PSA nadir

**Fig. 2:**
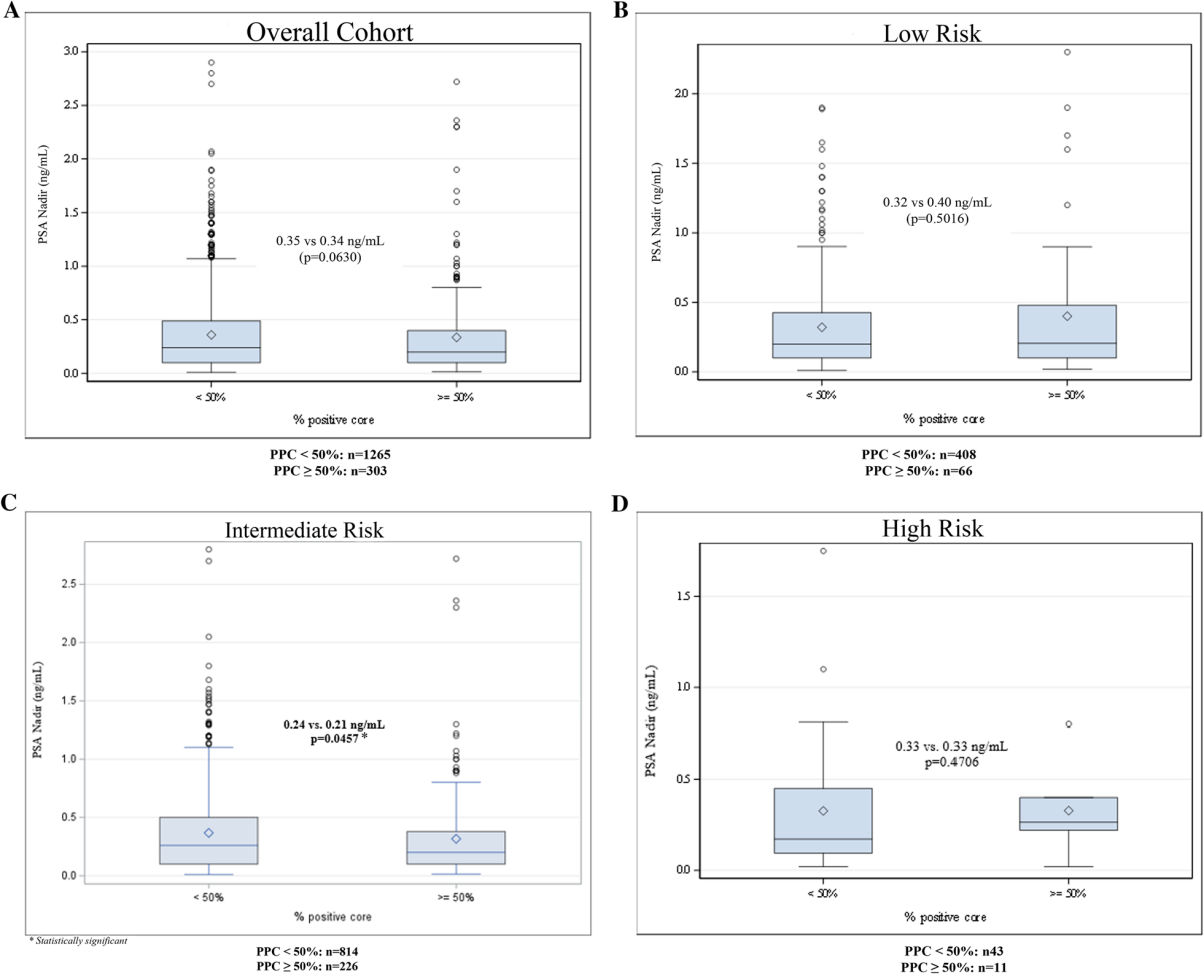
**A** Box plot of PSA nadir stratified by < 50% and ≥ 50% core involvement for overall cohort. **B** Box plot of PSA nadir stratified by < 50% and ≥ 50% core involvement for low-risk cohort. **C** Box plot of PSA nadir stratified by < 50% and ≥ 50% core involvement for intermediate-risk cohort. **D** Box plot of PSA nadir stratified by < 50% and ≥ 50% core involvement for high-risk cohort

In the overall cohort, high-volume core involvement was not associated with a higher PSA nadir. In fact, there was a non-significant trend towards higher PSA nadir with low-volume core involvement (0.35 vs 0.34 ng/mL, *p* = 0.063). Interestingly, when specifically analyzing the intermediate-risk cohort, low-volume biopsy core involvement demonstrated a significantly higher PSA nadir (0.35 vs 0.34 ng/mL, p = 0.004). Moreover, after adjusting for prostate CTV, there was still a statistically significant difference in PSA nadir (0.24 vs. 0.21, *p* = 0.046). The remaining low- and high-risk cohorts did not demonstrate any significant association with biopsy core involvement and PSA nadir. Box plots of PSA nadir and biopsy core involvement overall and stratified by risk group are displayed in Figs. 2A–D.

### Grade group weighted core involvement association with PSA nadir

**Fig. 3:**
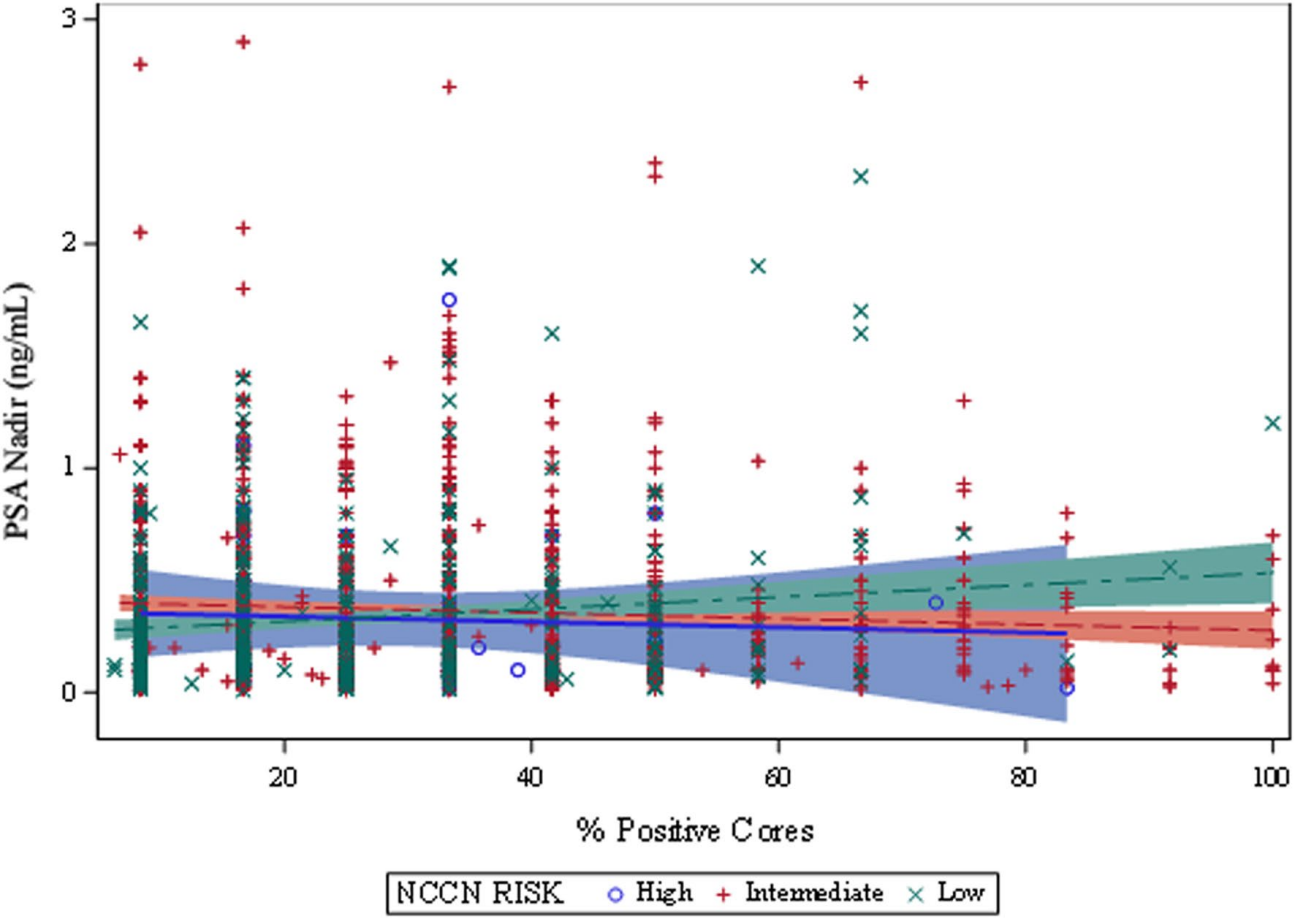
Grade group weighted core involvement association with PSA nadir for low, intermediate, and high risk groups

When weighted percent core involvement was analyzed as a continuous variable for the entire cohort, there was no significant association with PSA nadir using Spearman correlation coefficients (ρ = − 0.012, *p* = 0.617). However, when the intermediate-risk cohort was similarly analyzed, a significantly negative correlation was identified between higher weighted core involvement and PSA nadir (ρ = − 0.063, *p* = 0.049). No similar association was observed in the low- (ρ = 0.054, *p* = 0.249) or high-risk (ρ = − 0.0721, *p* = 0.667) cohorts. Figure 3 displays detailed Grade group weighted core involvement analysis.

### Core involvement association with oncologic outcomes

**Fig. 4:**
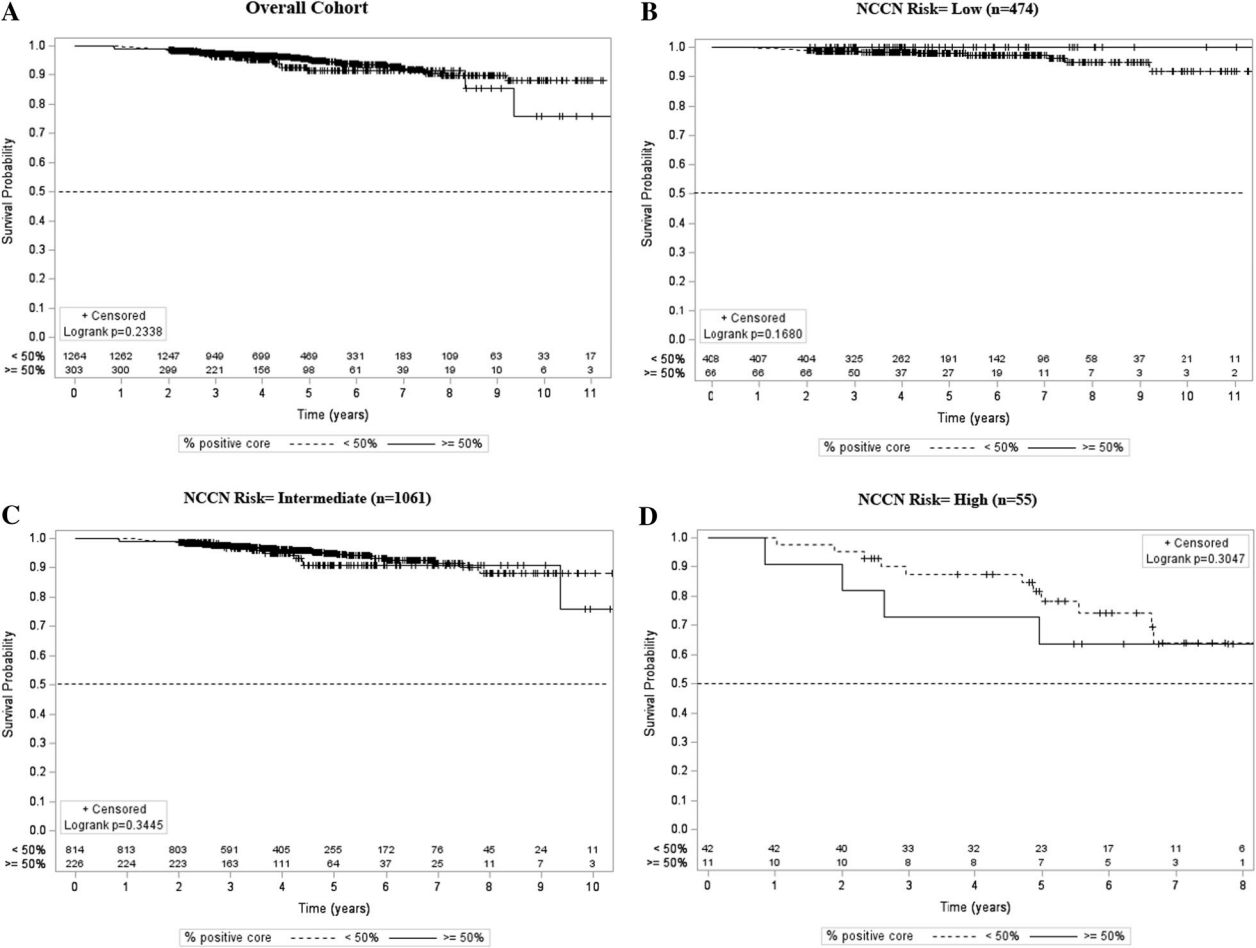
**A** Biochemical progression free survival stratified by < 50% and ≥ 50% biopsy core involvement for overall cohort. **B** Biochemical progression free survival stratified by < 50% and ≥ 50% biopsy core involvement for low risk cohort. **C** Biochemical progression free survival stratified by < 50% and ≥ 50% biopsy core involvement for intermediate cohort. **D** Biochemical progression free survival stratified by < 50% and ≥ 50% biopsy core involvement for high risk cohort

Core involvement was analyzed to determine its association with biochemical progression free survival (bPFS). With a median FU of 4.3 years (IQR: 3.1 to 6.2 years) for the entire cohort, there was no significant difference in time-to-failure between patients with high- versus low-volume biopsy core involvement (*p* = 0.234). In contrast, there was a significant association between time-to-failure and weighted total PC (HR = 1.02, 95% CI: 1.00–1.03, *p* = 0.012). However, after adjusting for prostate CTV this significance vanished (HR = 1.01, 95% CI: 0.99–1.03, *p* = 0.2562). Each NCCN risk subgroup was then analyzed in similar fashion independently, and there was again no significant association between bPFS and high- versus low-volume core involvement for low (*p* = 0.168), intermediate (*p* = 0.345), and high (*p* = 0.305) risk disease. Correspondingly, there was no significant association between weighted core involvement and each risk group when analyzed independently for low (HR = 0.94, 95% CI: 0.88–1.01, *p* = 0.115), intermediate (HR = 1.01, 95% CI: 0.99–1.03, *p* = 0.284), and high (HR = 1.02, 95% CI: 0.99–1.04, *p* = 0.141) risk disease. Of note, after adjusting for prostate CTV in the high-risk cohort, subjects with low-volume core involvement had a lower risk of progression (HR = 0.29, 95% CI: 0.09–0.99, *p* = 0.048). Multivariable analysis also did not demonstrate dichotomized PC involvement as associated with PCa failure. However, given the small number of patients with high-risk disease, the clinical significance of this finding is unclear. Figures 4A–D display detailed weighted core involvement analysis stratified by risk group.

## Discussion

In the present study with a median FU of over 4 years, we demonstrate no increased risk of bPFS in patients with high-volume core involvement who were treated for localized PCa with 5-fraction SBRT monotherapy. In fact, even when weighting biopsy core involvement by aggressiveness of histology, there was no correlation with increased risk of bPFS for intermediate risk disease. In general, high-volume core involvement was a relatively rare event, at least in a group of patients who did not receive ADT, occurring in 19% of our cohort. Moreover, high-volume core involvement was more commonly observed in patients who had intermediate-risk disease or smaller PV. Counterintuitively, within the intermediate cohort, PSA nadirs were found to be significantly higher in patients with low-volume core involvement. This may reflect PSA expression resilience in those with a higher volume of normal prostatic tissue.

Biopsy core involvement is inextricably linked to the type of biopsy performed and the volume of the prostate being biopsied [7, 8]. In a vacuum, a 12-core biopsy performed on a 20 cc prostate is naturally more reflective of the true cancer distribution versus a 12-core biopsy performed on a 200 cc prostate, as has been reflected in the literature [9]. This was manifested in our analysis, as high-volume core involvement was associated with smaller CTV. Prior research has demonstrated more extensive prostate biopsies (21 vs. 12 cores) correlate with a lower rate of identifying surprising unfavorable disease at prostatectomy [10]. The geometry of core involvement also plays a role, with contiguous biopsy core involvement having been demonstrated to correlate with extracapsular extension and seminal vesicle invasion [11]. As MRI-targeted biopsies become ubiquitous, the utility of capturing disease volume based on a 12-core blind biopsy is challenging to ascertain. Even the correct denominator used in calculating percentage core involvement when multiple targeted biopsies of a given ROI is calculated variably.

Prostate biopsy tumor quantitation has been explored using a variety of metrics in the literature including percent PC involvement, as in the present study, as well as greatest percentage of the most involved core and highest cumulative core length, amongst other metrics. Murgic et al. demonstrated the maximum involvement of a given biopsy core is the only prognostic factor for freedom from biochemical failure following conventionally fractionated radiation [12]. Percentage of positive prostate biopsy cores has been shown to be predictive on MVA of PSA outcome following surgical prostatectomy [13]. Finally, a review of 13 manuscripts determined prostate tumor quantitation using overall percentage or the greatest percentage of the most involved core was associated with clinical outcomes [14].

Within the low-risk realm, percentage core involvement makes a large impact in the decision making for those patients evaluated for active surveillance candidacy. The number of PC involved has been demonstrated as an independent risk factor on MVA for progression after first biopsy [15]. In general, core involvement appears to be predictive for upstaging low-risk cancers at the time of prostatectomy [16, 17]. Though, data regarding the value of prostate biopsy volume characteristics in low-risk PCa after treatment is conflicting with some reports highlighting its importance and others its insignificance [18–20].

Within the high-risk realm, increasing number of PC with high-grade cancer and > 50% PC involvement are predictive for unfavorable pathology identified at radical prostatectomy [21]. Maximum volume of high-grade (GS8-10) cancer per core has been shown on MVA as an independent predictor of final GS at pT stage [22]. Greatest percentage of a given involved biopsy core length has also been associated with adverse clinical outcomes following prostatectomy [23]. Higher cumulative PCa core length relative to the number of biopsy cores sampled is associated with identification of higher volume PCa at the time of prostatectomy, though it is unclear if this is confounded by PV [24].

A major limitation of the present study is the lack of differentiation of GS7 cancers into the more modern GG 2 versus 3 disease, which is a remnant of the retrospective evaluation of our pathology database. For the purposes of PSA nadir analysis, ADT was exclusionary and as such, we limit the generalizability of this dataset to only those patients who did not receive ADT in concert with SBRT. It is reasonable to hypothesize that high-volume core involvement would more commonly trigger ADT inclusion, and thus these patients were excluded from the present analysis. In addition, although the pathology reports were predominately based off 12 core prostate biopsies, some did include MRI-targeted biopsies. In such cases, a given region of interest with multiple biopsies taken was not counted as a single biopsy, as is now recommended in the modern version of the NCCN. The use of CTV as a surrogate for PV is also subject to error, particularly if large-volume seminal vesicles were included. Finally, the analysis of core involvement is also subject to selection bias given all patients received SBRT, and patients managed with other modalities of treatment were excluded.

## Conclusion

High-volume (≥ 50%) biopsy core involvement is not associated with an increased risk of PCa progression following 5-fraction SBRT for localized PCa. Longer FU is needed to confirm these findings. In the era of prostate SBRT and MRI-directed prostate biopsies, the use of high-volume core involvement as an independent increased risk factor should be revisited. Similarly, percentage of PC does not appear to be associated with higher recurrence in this relatively favorable risk group of patients. The conclusion likely does not hold for high-risk patients, as they were grossly underrepresented in this cohort.

### Supplementary Information

**Additional file 1. Supplementary Table 1A: **Association between continuous percent core involvement and patient, tumor, and treatment characteristics.

**Additional file 2. Supplementary Table 1B: **Association between percent dichotomized (i.e. <50% and ≥50%) core involvement and patient, tumor,  and treatment characteristics.

**Additional file 3. Supplementary Table 1C: **Percent positive cores in its continuous form represented for the multivariate Cox PH model.

**Additional file 4. Supplementary Table 1D: **Percent positive cores dichotomized as <50% vs ≥ 50% represented for the multivariate Cox PH model.

**Additional file 5. Supplementary Table 1E: **Time-to-nadir calculated as end of treatment date to updated PSA nadir date.

## Data Availability

Research data are stored in an institutional repository and will be shared upon request to the corresponding author.

## Abbreviations

Pca: Prostate cancer
FU: Follow up
SBRT: Stereotactic body radiation therapy
NCCN: National comprehensive cancer network
PC: Positive cores
ADT: Androgen deprivation therapy
GS: Gleason score
PSA: Prostate-specific antigen
BED: Biologically effective dose
IMRT: Intensity modulated radiation therapy
CT: Computed tomography
CTV: Clinical target volume
PTV: Prostate target volume
bPFS: Biochemical progression free survival
MVA: Multivariate analysis
PV: Prostate volume
