# Supplementary material for: High-volume prostate biopsy core involvement is not associated with an increased risk of cancer recurrence following 5-fraction stereotactic body radiation therapy monotherapy
Source: Radiat Oncol. 2024 Mar 4;19:29. doi: 10.1186/s13014-023-02397-z (PMC10913228; doi:10.1186/s13014-023-02397-z)
Supplement: Supplementary file 1 — Additional file 1. Supplementary Table 1A: Association between continuous percent core involvement and patient, tumor, and treatment characteristics. [file 13014_2023_2397_MOESM1_ESM.docx]

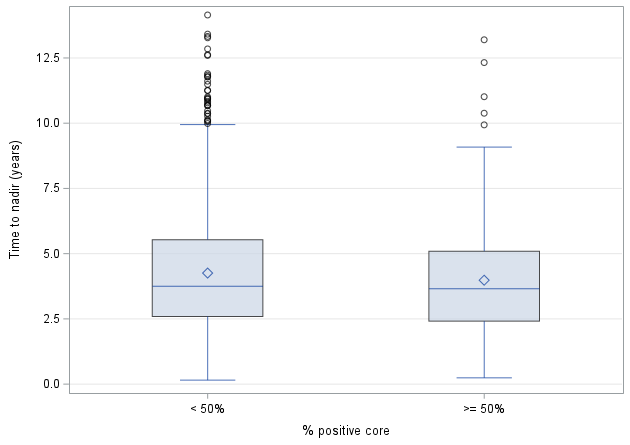
**Supplementary material:** Time-to-nadir calculated as end of treatment date to updated PSA nadir date

| **Analysis Variable: Time to nadir (years)** | | | | | | | | | | |
| --- | --- | --- | --- | --- | --- | --- | --- | --- | --- | --- |
| **% positive core** | **N Obs** | **N** | **Mean** | **Std Dev** | **Median** | **25th Pctl** | **75th Pctl** | **Minimum** | **Maximum** |  |
| **< 50%** | **1265** | 1265 | 4.26 | 2.32 | 3.75 | 2.59 | 5.54 | 0.15 | 14.15 |  |
| **>= 50%** | **303** | 303 | 3.98 | 2.15 | 3.66 | 2.41 | 5.10 | 0.24 | 13.20 |  |
