# Supplementary material for: High-volume prostate biopsy core involvement is not associated with an increased risk of cancer recurrence following 5-fraction stereotactic body radiation therapy monotherapy
Source: Radiat Oncol. 2024 Mar 4;19:29. doi: 10.1186/s13014-023-02397-z (PMC10913228; doi:10.1186/s13014-023-02397-z)
Supplement: Supplementary file 2 — Additional file 2. Supplementary Table 1B: Association between percent dichotomized (i.e. <50% and ≥50%) core involvement and patient, tumor, and treatment characteristics. [file 13014_2023_2397_MOESM2_ESM.docx]

**Supplementary Table 1A:** Association between continuous percent core involvement and patient, tumor, and treatment characteristics.

|  | **Percent core involvement** | **p-value** |
| --- | --- | --- |
| **Age at treatment*** | rho = 0.002 | 0.945 |
| **Initial PSA (mg/mL)*** | rho = 0.015 | 0.569 |
| **PSA (mg/mL)**:** |  | 0.701 |
| <10 | 30.1 ± 18.9 |  |
| *[10 – 20]* | 29.3 ± 19.8 |  |
| *>20* | 25.0 ± 8.3 |  |
| **Gleason Scores**:** |  | <0.0001* |
| 6 | 24.7 ± 16.6 |  |
| *7* | 33.0 ± 19.7 |  |
| *8* | 29.1 ± 15.9 |  |
| *9* | 44.4 ± 34.7 |  |
| **NCCN Risk**:** |  | <0.0001* |
| Low | 30.0 ± 17.3 |  |
| *Intermediate* | 32.0 ± 19.7 |  |
| *High* | 25.6 ± 17.0 |  |
| **Prostate CTV*** | rho = -0.140 | <0.0001* |

** Data presented as Spearman correlation coefficient and corresponding p-value.*

*** The groupings for categorical PSA, Gleason score, and NCCN risk were each compared separately with respect to continuous percent core involvement using analysis of variance for 3 or more groups. Data are presented as mean ± standard deviation.*
