# Supplementary material for: High-volume prostate biopsy core involvement is not associated with an increased risk of cancer recurrence following 5-fraction stereotactic body radiation therapy monotherapy
Source: Radiat Oncol. 2024 Mar 4;19:29. doi: 10.1186/s13014-023-02397-z (PMC10913228; doi:10.1186/s13014-023-02397-z)
Supplement: Supplementary file 3 — Additional file 3. Supplementary Table 1C: Percent positive cores in its continuous form represented for the multivariate Cox PH model. [file 13014_2023_2397_MOESM3_ESM.docx]

**Supplementary Table 1B:** Association between percent dichotomized (i.e. <50% and ≥50%) core involvement and patient, tumor and treatment characteristics

|  | **<50%** | **≥50%** | **p-value** |
| --- | --- | --- | --- |
| **Age at treatment (mean ± SD)** | 66.0 ± 7.2 | 65.6 ± 7.5 | 0.4343 |
| **Initial PSA (mg/mL)** | 5.9 [4.6, 4.9] | 6.1 [4.7, 7.8] | 0.7288 |
| **PSA (mg/mL):** |  |  | 0.5339 |
| *<10* | 1030 (85.90%) | 250 (88.34%) |  |
| *[10 – 20]* | 166 (13.84%) | 33 (11.66%) |  |
| *>20* | 3 (0.25%) | 0 (0.00%) |  |
| **Gleason Scores:** |  |  | 0.0001* |
| *6* | 457 (38.12%) | 71 (25.09%) |  |
| *7* | 713 (59.47%) | 206 (72.79%) |  |
| *8* | 27 (2.25%) | 5 (1.77%) |  |
| *9* | 2 (0.17%) | 1 (0.35%) |  |
| **NCCN Risk:** |  |  | 0.0053 |
| *Low* | 32 (2.67%) | 6 (2.12%) |  |
| *Intermediate* | 773 (64.47%) | 211 (74.56%) |  |
| *High* | 394 (32.86%) | 66 (23.32%) |  |
| **Prostate CTV** | 78.7 [63.0, 102.1] | 70.0 [56.0, 90.1] | <0.0001* |

*Core involvement was dichotomized as <50% and ≥50%. Groups were compared using the two sample-test for age at treatment and presented as mean ± SD. The Mann-Whitney test was used to compare the two groups for initial PSA and prostate CTV and data was reported as median [25^th^, 75^th^ percentiles]. Fisher’s exact test was used to compare the groups for categorical Gleason Score and NCCN risk and presented as frequency (%).*
