# Supplementary material for: High-volume prostate biopsy core involvement is not associated with an increased risk of cancer recurrence following 5-fraction stereotactic body radiation therapy monotherapy
Source: Radiat Oncol. 2024 Mar 4;19:29. doi: 10.1186/s13014-023-02397-z (PMC10913228; doi:10.1186/s13014-023-02397-z)
Supplement: Supplementary file 4 — Additional file 4. Supplementary Table 1D: Percent positive cores dichotomized as <50% vs ≥ 50% represented for the multivariate Cox PH model. [file 13014_2023_2397_MOESM4_ESM.docx]

**Supplementary Table 1C:** Percent positive cores in its continuous form represented for the multivariate Cox PH model

| **Covariate** |  | **Hazard Ratio** | **95% Wald Confidence Limits** | | **Pr > ChiSq** |
| --- | --- | --- | --- | --- | --- |
| **Age at TX** | *unit=5* | 0.78 | 0.65 | 0.95 | 0.013 |
| **Initial PSA** | *unit=1* | 1.18 | 1.10 | 1.27 | <0.001 |
| **% Positive Cores** | *unit=5* | 0.97 | 0.90 | 1.04 | 0.371 |
| **Prostate CTV** | *unit=10* | 0.98 | 0.89 | 1.07 | 0.609 |
| **NCCN RISK** | *High* | 0.26 | 0.02 | 3.83 | 0.324 |
|  | *Intermediate* | 0.45 | 0.11 | 1.81 | 0.261 |
|  | *Low* | *[reference]* | | | |
| **Total # Cores** | *unit=1* | 0.93 | 0.77 | 1.12 | 0.427 |
| **Gleason Score** | *6* | *[reference]* | | | |
|  | *7* | 3.23 | 0.90 | 11.60 | 0.073 |
|  | *8* | 26.73 | 2.02 | 353.88 | 0.013 |
|  | *9* | 12.73 | 0.76 | 212.27 | 0.077 |
