# Supplementary material for: High-volume prostate biopsy core involvement is not associated with an increased risk of cancer recurrence following 5-fraction stereotactic body radiation therapy monotherapy
Source: Radiat Oncol. 2024 Mar 4;19:29. doi: 10.1186/s13014-023-02397-z (PMC10913228; doi:10.1186/s13014-023-02397-z)
Supplement: Supplementary file 5 — Additional file 5. Supplementary Table 1E: Time-to-nadir calculated as end of treatment date to updated PSA nadir date. [file 13014_2023_2397_MOESM5_ESM.docx]

**Supplementary Table 1D:** Percent positive cores dichotomized as <50% vs. ≥ 50% represented for the multivariate Cox PH model

| **Covariate** |  | **Hazard Ratio** | **95% Wald Confidence Limits** | | **Pr > ChiSq** |
| --- | --- | --- | --- | --- | --- |
| **Age at TX** | *unit=5* | 0.79 | 0.66 | 0.96 | 0.017 |
| **Initial PSA** | *unit=1* | 1.18 | 1.10 | 1.26 | <.0001 |
| **% Positive Cores** | *< 50%* | 1.03 | 0.51 | 2.10 | 0.929 |
|  | ≥ *50%* | *[reference]* | | | |
| **Prostate CTV** | *unit=10* | 0.98 | 0.90 | 1.08 | 0.688 |
| **NCCN RISK** | *High* | 0.30 | 0.02 | 4.45 | 0.385 |
|  | *Intermediate* | 0.47 | 0.12 | 1.91 | 0.293 |
|  | *Low* | *[reference]* | | | |
| **Total # Cores** | *unit=1* | 0.93 | 0.77 | 1.13 | 0.465 |
| **Gleason Score** | *6* | *[reference]* | | | |
|  | *7* | 2.96 | 0.82 | 10.63 | 0.097 |
|  | *8* | 21.74 | 1.69 | 279.80 | 0.018 |
|  | *9* | 8.21 | 0.55 | 122.95 | 0.128 |
